# Identification of variants and therapeutic epitopes in HPV-33/HPV-58 E6 and E7 in Southwest China

**DOI:** 10.1186/s12985-019-1168-y

**Published:** 2019-05-28

**Authors:** Jiaoyu He, Yasi Yang, Zuyi Chen, Yang Liu, Shanfei Bao, Yun Zhao, Xianping Ding

**Affiliations:** 1 Key Laboratory of Bio-Resource and Eco-Environment of Ministry of Education, College of Life Sciences, Sichuan University, Chengdu, 610065 Sichuan People’s Republic of China; 2 Bio-resource Research and Utilization Joint Key Laboratory of Sichuan and Chongqing, Chongqing, People’s Republic of China; 3 Chongqing Innovation Academy of Characteristic Bioindustry Technology, Chongqing, People’s Republic of China; 4 Center of Growth, Metabolism and Aging, Key Laboratory of Bio-Resource and Eco-Environment of Ministry of Education, College of Life Sciences, Sichuan University, Chengdu, People’s Republic of China

**Keywords:** Human papillomavirus, Cervical Cancer, HPV-33, HPV-58, E6, E7, Human leukocyte antigen, Epitopes prediction, Bioinformatics, Southwest China

## Abstract

**Background:**

Human papillomavirus (HPV) E6 and E7 oncoproteins play a crucial role in HPV-related diseases, such as cervical cancer, and can be used as ideal targets for therapeutic vaccines. Human leukocyte antigen (HLA) participates in the immune response to block HPV infection and invasion by its target/recognition function. HPV-33 and HPV-58 are highly prevalent among Chinese women. Therefore, it is of great significance to study the *E6* and *E7* region-specific gene polymorphisms of HPV-33 and HPV-58 in Southwest China and to identify ideal epitopes for vaccine design. Both HPV-33 and HPV-58 belong to α-9 genus HPV and are highly homologous, so their correlations are included in our research.

**Methods:**

To study the *E6* and *E7* variations and polymorphisms of HPV-33 and HPV-58 in Southwest China, we collected samples, extracted and sequenced DNA, and identified variants. Nucleotide sequences were translated into amino acids by Mega 6.0 software. The physical/chemical properties, amino acid-conserved sequences and secondary structure of protein sequences were analysed by the Protparam server, ConSurf server and PSIPRED software. The T and B cell epitopes of the E6/E7 reference and variant sequences in HPV-33 and HPV-58 were predicted by the Immune Epitope Database (IEDB) analysis server and the ABCpred server, respectively.

**Results:**

Five and seven optimal HLA-I restricted T cell epitopes were selected from HPV-33 and HPV-58 E6, respectively, and these optimal epitopes are mainly located in _41-58_EVYDFAFADLTVVYREGN of HPV-33 E6 and _40-60_SEVYDFVFADLRIVYRDGNPF of HPV-58 E6. Six optimal HLA-I-restricted T cell epitopes were selected from HPV-33 and HPV-58 E7, and these epitopes are mainly located in _77-90_RTIQQLLMGTVNIV of HPV-33 E7 and _78-91_RTLQQLLMGTCTIV of HPV-58 E7.

**Conclusions:**

HPV-33/HPV-58 *E6/E7* gene polymorphisms and T/B cell epitopes of their reference and variant sequences were studied, and candidate epitopes were selected by bioinformatics techniques for therapeutic vaccine design for people in Southwest China. This study was the first to investigate the correlation of epitopes between HPV-33 and HPV-58. After experimental validation, these selected epitopes will be employed to induce a wide range of immune responses in heterogeneous HLA populations.

**Electronic supplementary material:**

The online version of this article (10.1186/s12985-019-1168-y) contains supplementary material, which is available to authorized users.

## Background

Human papillomavirus (HPV) is a double-stranded type of DNA virus of approximately 8 kb in size and is widely spread in humans [1]. HPV infection not only causes warts on the human skin and mucosa but also leads to the occurrence of malignant tumours [2]. In most developing countries, cervical cancer is the most common type of cancer in women, and 99.7% of cervical cancer patients have been diagnosed with HPV infection [3, 4]. Based on pathogenicity, HPV types are classified into high risk and low risk. Strong molecular epidemiological evidence indicates that persistent infection with high-risk HPV is a major cause of invasive cervical cancer and type II/III cervical intraepithelial neoplasia, and HPV-16, 18, 31, 33, 35, 39, 56, 58, and 59 are the common high-oncogenic risk types [5, 6]. Exogenous vaginal condyloma mutations and type I cervical intraepithelial neoplasia lesions in the crissum/lower genital tract are predominantly caused by low-risk HPV, such as HPV-6, 11, 40, 42, 43, 44, 54, 61, 70, 72, and 81 [3].

E6 and E7 proteins are the main virus-transforming proteins of high-risk HPV that participate in inducing cell proliferation and causing human epithelial cell immortalization and transformation [7]. E6 proteins bind to the ligase E6AP to form a complex that can inactivate the important tumour suppressor protein p53. The primary targets of E7 proteins are the retinoblastoma (Rb) family tumour suppressor proteins [8]. E6 and E7 play a key role in the occurrence and development of cervix precancerous lesions and invasive cervical cancer [9]. Thus, HPV E6 and E7 proteins are ideal targets for diagnostic detection and therapeutic vaccine design.

Human leukocyte antigen (HLA) has a self-recognition function to regulate the body’s immune response and control HPV infection and virus removal by presenting antigen proteins [9–11]. HLA antigens distributed in nucleated cells are composed of numerous alleles with different frequencies and are primarily divided into three types: HLA-I, HLA-II, and HLA-III [11–14]. HLA-I recognizes and stimulates CD8^+^ cytotoxic T lymphocytes (CTLs). HLA-II assists in identification of exogenous antigen and stimulates CD4^+^ helper T lymphocytes (HTLs). In cell-mediated immune responses, CTLs are considered the major eradicators of both HPV-infected and cervical cancer cells [15]. CD8^+^CTLs activated after proliferation can directly kill tumour cells or secrete cytokines to inhibit tumour cells. Lymphatic factors produced by the activated CD4^+^ HTLs enhance the function of CTLs and natural killer (NK) cells and activate macrophages or other antigen-presenting cells (APC), which have antitumour effects [16]. CTL epitopes typically have 8–11 amino acids and bind to the cleft of various HLA-I molecules by embedding in the peptide sequence [16, 17]. The specificity of epitopes is determined by the antigen peptide, which is composed of specific amino acids in foreign proteins (e.g., viral protein). Polymorphisms and distribution characteristics of HLA alleles and HPV genes are very important in immune recognition [12, 18]. B cells play an important role in HPV-related cancer immunotherapy and responses to cervical epithelial neoplasm and invasive cancer caused by HPV [17].

The treatment of HPV-related diseases by antigen epitopes was previously proposed [16]. Therefore, the therapeutic significance of T lymphocyte and B lymphocyte epitopes must be taken seriously. Virus mutations may cause differences in immune response and oncogenic potential [17]. Epidemiological studies have shown that the prevalence of HPV types and variants vary in different geographic areas and populations, and the same HPV type contains different mutations in different regions and populations [18]. HPV-33 and HPV-58 belong to the α-9 genus, which contains almost all carcinogenic types. Given the similarity between HPV-33 and HPV-58 and the importance of E6/E7, *E6/E7* gene diversity of HPV-33/HPV-58 in Southwest China was chosen as the subject of this study [19]. To improve vaccine accuracy and late-stage experiment effectiveness, the relationship of antigen epitopes between HPV-33 and HPV-58 E6/E7 were investigated by means of bioinformatics, and candidate T-lymphocyte/B-lymphocyte antigen epitopes were selected for vaccine design.

## Materials and methods

### Sample collection source

Samples were collected from hospitals in Sichuan and Chongqing, including the Angel of Maternal and Child Health Hospital and the Reproductive Health Research Center of Chengdu. This study was approved by the Ethics Education and Research Committee of Sichuan University and the Ethics Committee of Sichuan University (Sichuan, China). A total of 16,793 cervical specimens were collected from January 1, 2009 to December 31, 2015. All methods were performed in accordance with the relevant guidelines and regulations. Specimens were collected from participants using cervical swabs and stored at − 20 °C in buffer solution (9 g NaCl, 10 g C_6_H_5_CO_2_Na, 1 L H_2_O).

### Sample collection standard

Samples were collected from patients undergoing cervical screenings, histology, and cytology evaluations for cervical diseases (age range 16 ± 87 years, average age 33.02, median age 29). Subjects over 14 years old with visible cervical lesions or HPV-related diseases (e.g., cervicitis, cervical intraepithelial neoplasia, and cervical cancer) were included [20].

### Human papillomavirus DNA extraction

HPV DNA was extracted and evaluated using the Human Papillomavirus Genotyping Kit For 23 Types (Yaneng Bio, Shenzhen, China) according to the manufacturer’s guidelines.

### PCR amplification and identification of variants

Primers were designed according to the HPV-33/HPV-58 reference sequence. HPV-33/HPV-58 complete *E6/E7* genes were amplified by polymerase chain reaction (PCR) using a thermal cycler (Longgene, Hangzhou, China). Primers for HPV-33 *E6* and *E7* were as follows: 5′-AAAAAAGTAGGGTGTAACCGA-3′ and 5′-TGCCACTGTCATCTGCTGT-3′ (melting temperature 54 °C) as well as 5′-ACGGTGCATATATAAAGCAAACATT-3′ and 5′-CTTCTACCTCAAACCAACCAGTACA-3′ (melting temperature 60 °C) when a second round of amplification was needed. HPV-58 *E6* and *E7* fragments were amplified using specific primers described previously [21]. The total PCR mixture was 50 μL, including 5 μL of extracted DNA (10 ± 100 ng), 200 μmoL of MgCl_2_ and dNTPs, 2 U of Pfu DNA polymerase (Sangon Biotech, Shanghai, China), and 0.25 μmoL of each primer. Conditions were as follows: 95 °C (10 min); 35 cycles of 94 °C (50 s), 54 °C (60 s) (different for each gene), 72 °C (60 s); and a final step of 72 °C for 7 min. PCR amplification products were stained with Gene-Green nucleic acid dyes and visualized on 2% agarose gels under ultraviolet light WFH-202. Target products were sequenced at Sangon Biotech (Shanghai, China). Data were confirmed by performing the PCR amplification and sequence analysis at least twice.

### Sequence and structural analysis

Data were analysed by SPSS version 19 (Armonk, IBM, New York, USA). Pearson chi-square test was employed to confirm the results. *P* < 0.05 was considered statistically significant. Compared with the reference sequence, mutations with a frequency ≥ 10% were considered as major mutations [17]. Mega 6.0 software was used to translate nucleotide sequences into amino acids [22]. The Protparam server was used to predict physical and chemical properties of protein sequences, amino acid composition, molecular weight, theoretical isoelectric points, proportion of strong alkali/acid, hydrophobic, and amino acid polarity [23]. Moreover, the ConSurf server was used to identify amino acid-conserved sequences and secondary structures [23]. The PSIPRED server is a very strict cross-validation technique evaluation method that simply and accurately predicts protein secondary structure [23].

### HLA allele retrieval and analysis

According to the average frequency of HLA alleles from the major histocompatibility complex database (dbMHC) in the Chinese population [24], 13 HLA-I and 6 HLA-II alleles were selected in our study (Tables 1 and 2).

**Table 1:** Average frequency of HLA-I alleles (> 5%) across the Chinese population

Alleles	Average frequency	Alleles	Average frequency	Alleles	Average frequency
HLA-A*11:01	27.7%	HLA-B*40:01	14.9%	HLA-C*01:02	16.9%
HLA-A*24:02	17.2%	HLA-B*46:01	11.5%	HLA-C*03:04	12.8%
HLA-A*33:03	11.5%	HLA-B*58:01	8.9%	HLA-C*08:01	12.6%
HLA-A*02:01	5.3%	HLA-B*13:01	8.2%	HLA-C*03:02	8.7%
		HLA-B*15:02	7.1%		

Note: Lacking information about the frequency in Chinese individuals, the data from Asian individuals were used

**Table 2:** Average frequency of HLA-II alleles (> 5%) across the Chinese population

Alleles	Average frequency	Alleles	Average frequency	Alleles	Average frequency
DRB1*14:01	13.4%	DRB1*15:02	5.6%	DQB1*02:01	9.3%
DRB1*12:02	11.4%	DQB1*05:01	10.9%	DPB1*04:01	17.1%

Note: Lacking information about the frequency in Chinese individuals, the data from Asian individuals were used

### Epitope prediction

#### T cell antigen epitope

T cell epitopes of HPV-33/HPV-58 E6/E7 reference and variant sequences were predicted by Immune Epitope Database (IEDB) analysis server. According to the method recommended by IEDB, the lower percentile rank (PR) of antigen epitopes represented a better affinity. Thus, we selected candidate epitopes based on PR [23]. In our study, we used the IEDB recommended methods to predict the epitopes against 13 HLA-I alleles (Table 1) and 6 HLA-II alleles (Table 2). In prediction of HLA-I-restricted epitopes, epitope length was set to “All Length”, which contained the lengths of 8, 9, 10, 11, 12, 13 and 14. Peptides with mean PR < 1.0 were selected for analysis. In the prediction of HLA-II-restricted epitopes, all peptides with PR < 5.0 were selected for further analysis.

#### B cell antigen epitope

The ABCpred server was used to predict B cell antigen epitopes of HPV-33 and HPV-58 E6/E7 reference and variant sequences according to the default parameters [23]. The higher the predicted score, the better the epitope’s affinity.

### Data availability

The datasets generated during the current study are available in GenBank (accession codes for HPV-33 and HPV-58 *E6/E7* variant sequences are KX354744-KX354775).

## Results

### Mutation analysis of *E6* and *E7*

#### HPV-33 *E6* and *E7* polymorphism analysis

A total of 216 HPV-33 samples were sequenced. Data showed that the total length of the HPV-33 *E6* Open Reading Frame (ORF) was 450 bp and that of *E7* was 294 bp. Compared with the HPV-33 *E6/E7* reference sequence (GenBank: M12732.1), mutations in HPV-33 *E6* (450 bp) and HPV-33 *E7* (297 bp) nucleotide sequences are presented in Table 3. Among all samples, 76 (35.19%) *E6* sequences and 96 (44.45%) *E7* sequences have nucleotide mutations. In these mutant *E6* sequences, eight nucleotide mutations were observed, including six non-synonymous mutations and two synonymous mutations. Non-synonymous mutations include A213C, G329C, A364C, A387C, A446G, and G542 T, leading to the amino acids substitution of K35 N, S74 T, N86H, K93 N, Q113R and R145I, respectively. Among them, the most common mutations occurred at the 213th and 387th site of *E6* sequence with a 19.44% mutation rate, and both amino acid substitutions are from lysine to aspartate. Finally, seven HPV-33 *E6* variant types were determined. In the *E7* mutant sequences, four single nucleotide changes were identified, which were all non-synonymous mutations, including G658C, C706T, C706A, and A862T that lead to the amino acids substitution of S29 T, A45V, A45E and Q97L, respectively. The most common mutation was C706T (A45V) with a 16.67% mutant rate. In addition, seven HPV-33 *E7* variant sequence types were determined.

**Table 3:** Nucleotide variations and amino acid substitutions in HPV-33 *E6* and *E7*

HPV-33	*E6*								*E7*			
S. No.	M1	M2	M3	M4	M5	M6	M7	M8	M1	M2	M3	M4
Position	213	273	329	364	387	446	542	549	658	706	706	862
Ref	A	A	G	A	A	A	G	T	G	C	C	A
Normal	–	–	–	–	–	–	–	–	–	–	–	–
A	–	–	–	–	–	–	–	–	–	–	A	–
T	–	–	–	–	–	–	T	–	–	T	–	T
G	–	G	–	–	–	G	–	C	–	–	–	–
C	C	–	C	C	C	–	–	–	C	–	–	–
Frequency	42	17	7	16	42	16	33	25	16	36	27	19
% of Samples	19.44	7.87	3.24	7.41	19.44	7.41	15.28	11.57	7.4	16.67	12.5	8.8
Amino acid Mutation	K35N	–	S74T	N86H	K93N	Q113R	R145I	–	S29T	A45V	A45E	Q97L
Secondary structure	C	S	H	C	C	H	C	C	C	C	C	C

Note: In the row of ‘S. No’, M refers to mutation. The row of ‘Ref’ refers to the base in reference sequence. In the row of ‘Secondary structure’, C refers to coil, S refers to β-sheet, and H refers to α-helix

#### Analysis of HPV-58 *E6* and *E7* polymorphisms

In this study, 405 HPV-58 samples were sequenced, and the mutations in HPV-58 *E6* (450 bp) and HPV-58 *E7* (297 bp) are presented in Table 4. Compared with the HPV-58 *E6/E7* reference sequence, 356 (87.90%) *E6* variant sequences and 326 (80.50%) *E7* variant sequences were detected. In these *E6* variant sequences, eight variant types and eight nucleotide mutations were found, including 4 non-synonymous mutations and 4 synonymous mutations. Non-synonymous mutations were G203C, C367A, A388C and G543A, resulting in the amino acids substitution of E32Q, D86E, K93N and R145K, respectively. A338C (K93N) is the most common non-synonymous mutation with a 27.41% mutation rate. In 326 *E7* variant sequences, twelve variant types were determined, and thirteen nucleotide mutations were found, including 10 non-synonymous mutations and 3 synonymous mutations. Non-synonymous mutations include G599A, C632T, G694A, C755A, G760A, G761A, A763G, A793G, C801A, and T803C, which lead to the amino-acid substitutions of R9K, T20I, G41R, T61N, G63S, G63D, T64A, T74A, D76E and V77A, respectively. G761A (G63D) was the most common non-synonymous mutation with a 40.25% mutation rate.

**Table 4:** Nucleotide variations and amino acid substitutions in HPV-58 E6 and E7

HPV-58	*E6*								*E7*												
S. No.	M1	M2	M3	M4	M5	M6	M7	M8	M1	M2	M3	M4	M5	M6	M7	M8	M9	M10	M11	M12	M13
Position	187	203	259	307	367	388	395	543	599	632	694	726	744	755	760	761	763	793	798	801	803
Ref	C	G	A	C	C	A	T	G	G	C	G	T	T	C	G	G	A	A	C	C	T
Normal	–	–	–	–	–	–	–	–	–	–	–	–	–	–	–	–	–	–	–	–	–
A	–	–	–	–	A	–	–	A	A	–	A	–	–	A	A	A	–	–	–	A	–
T	T	–	–	T	–	–	–	–	–	T	–	–	–	–	–	–	–	–	T	–	–
G	–	–	G	–	–	–	–	–	–	–	–	–	G	–	–	–	G	G	–	–	–
C	–	C	–	–	–	C	C	–	–	–	–	C	–	–	–	–	–	–	–	–	C
Frequency	7	1	2	237	1	111	1	16	13	74	162	7	319	26	74	163	26	1	1	1	82
% of Samples	1.73	0.25	0.49	58.52	0.25	27.41	0.25	3.95	3.21	18.27	40.00	1.73	78.76	6.42	18.27	40.25	6.42	0.25	0.25	0.25	20.25
Amino acid Mutation	–	E32Q	–	–	D86E	K93N	–	R145K	R9K	T20I	G41R	–	–	T61N	G63S	G63D	T64A	T74A	–	D76E	V77A
Secondary structure	S	C	S	H	C	C	C	C	C	C	C	C	S	C	C	C	C	H	H	H	H

Note: In the row of ‘S. No’, M refers to mutation. The row of ‘Ref’ refers to the base in reference sequence. In the row of ‘Secondary structure’, C refers to coil, S refers to β-sheet, and H refers to α-helix

### Structural analysis

#### HPV-33 E6 and E7 structural analysis

In this study, amino acids composition analysis of HPV-33 E6 and E7 was performed, and the residue numbers of HPV-33 E6 and E7 were 149 and 97, respectively. In the reference sequence of HPV-33 E6, Arg (10.10%) and Leu (10.10%) represented the highest residue proportion followed by Glu (8.10%) and Cys (6.70%). In reference sequence of E7, the top 3 residues with the highest content were Thr (11.30%), Leu (10.30%) and Asp (9.30%). An additional file shows more details [see Additional file 1: Table S1 and S2].

The secondary structure prediction of HPV-33 E6 showed that E6 reference contained 27.5% helix, 20.8% sheet, and 51.7% coil, whereas the E6 variant was composed of 28.2% helix, 16.1% sheet, and 55.7% coil. The result suggests that the mutation of E6 mainly affects the secondary structure of the residues 87–88 and 132–134, resulting in an increase of coil and a decrease of β-sheet. The prediction results of E7 revealed that E7 reference consists of 12.4% helix, 16.5% sheet, and 71.1% coil, and the substitutions had no influence on its secondary structure (Table 5). Details are shown in Additional file 2: Figure S1 and S2.

**Table 5:** Secondary structure prediction of HPV-33/58 E6 and E7

Target protein	Sequence type	No. helix/Helices^a^	No. sheet/Sheets^b^	No. coil/Coils^c^	Accessibility type	
					Burred	Exposed
HPV-33 E6	Reference	41/27.52%	31/20.80%	77/51.68%	50/33.56%	99/66.44%
	Variant	42/28.19%	24/16.11%	83/55.70%	49/32.89%	100/67.11%
HPV-58 E6	Reference	41/27.52%	25/16.78%	83/55.70%	48/32.22%	101/67.78%
	Variant	42/28.19%	25/16.78%	82/55.03%	48/32.22%	101/67.78%
HPV-33 E7	Reference	12/12.37%	16/16.50%	69/71.13%	27/27.84%	70/72.16%
	Variant-1	11/11.34%	16/16.50%	70/72.16%	30/30.93%	67/69.07%
	Variant-2	11/11.34%	16/16.50%	70/72.16%	28/28.87%	69/71.13%
HPV-58 E7	Reference	12/12.24%	17/17.35%	69/70.41%	28/28.57%	70/71.43%
	Variant-1	12/12.24%	17/17.35%	69/70.41%	26/26.53%	72/73.47%
	Variant-2	12/12.24%	17/17.35%	69/70.41%	26/26.53%	72/73.47%

Note: In the sequence type column, ‘reference’ and ‘variant’ indicate the reference sequence and variant sequence of the corresponding protein, respectively. ‘No. helix/Helices^a^’, ‘No. sheet/Sheets^b^’ and ‘No. coil/Coils^c^’ represent the number/ratio of residues located in helix, sheet and coil, respectively. ‘Burred’ and ‘Exposed’ represent the number/ratio of residues burred in protein and exposed at the surface, respectively

#### HPV-58 E6 and E7 structural analysis

Our analysis showed that HPV-58 E6 and E7 were composed of 149 and 98 residues, respectively. In the E6 reference sequence, Arg (10.70%) and Leu (10.70%) represented the highest proportion of residues followed by Glu (7.40%), Cys (7.40%) and Lys (7.40%). In the E7 reference sequence, the top 3 residues with the highest content were Thr (13.30%), Leu (10.20%) and Cys (9.20%). More details are shown in Additional file 1: Table S3 and S4.

Secondary structure prediction of HPV-58 E6 demonstrated that E6 reference is composed of 27.5% helix, 16.8% sheet, and 55.7% coil, similarly, E6 variant is 28.2, 16.8, and 55%. The above fact indicated that the substitutions merely cause the change of secondary structure in the residue 87. The prediction results of E7 illustrated that both E7 and its variant possess the similar secondary structure (12.2% helix, 17.3% sheet, 70.5% coil), suggesting that the substitutions are less important for secondary structure (Table 5). More details are shown in Additional file 2: Figure S3 and S4.

### T cell epitope prediction

#### T cell epitope prediction for HPV-33 E6 and E7

Based on the principle of epitope selection described in the methods section, we selected 171 and 180 HLA-I-restricted epitopes from the HPV-33 E6 reference and variant sequence, respectively (see Additional file 3: Table S5). First, we analysed the effect of mutations on epitope affinity based on these selected epitopes. Some mutations had obvious effects on the affinity of predicted epitopes. For example, replacement of N86H in _77-88_RHYNYSVYGNTL for HLA-A*24:02 and K35N in _25-37_NIELQCVECKKPL for HLA-B*40:01 resulted in a lower PR, which reflects a better binding affinity. Replacement of N86H and K93N in the ideal epitope _82-93_SVYGNTLEQTVK for HLA-A*11:01 resulted in PR greater than 1.0, which indicates low affinity. Second, to select the optimal predictions for therapeutic vaccine, we integrated the predicted epitopes without mutation sites into continuous segments (Table 6). Predicted epitopes occurred most frequently in the 36–72 segment, and the predicted epitopes with lowest PR (0.1) frequently appeared in the 41–58 segment. The same analysis was used to HPV-33 E7. In total, 9 and 9/8 potential epitopes were selected from reference and variant-1/variant-2 sequences, respectively (see Additional file 3: Table S6). Among the non-synonymous mutations found in the E7 variant sequences, only A45V (A45E) appeared in the predicted epitopes. Moreover, the substitution of A45V and A45E resulted in a PR of _42-52_DGQAQPATADY and _43-52_GQAQPATADY for HLA-B*15:02 greater than 1.0, respectively. After the integration of predicted epitopes without mutation sites, the epitopes appeared more frequently in the 77–90 segment (Table 6), and the epitope with the lowest PR (0.1) was in this segment.

**Table 6:** Distribution of predicted T cell epitopes

Target protein	Distribution of HLA-I epitopes (epitope’s number)	Distribution of HLA-II epitopes (epitope’s number)
HPV-33 E6	15~29 (8)	
	36~72 (97)	38~74 (25)
	75~85 (2)	
	96~110 (10)	
	124~144 (16)	
HPV-33 E7	52~66 (3)	3~25 (22)
	77~90 (5)	46~96 (27)
HPV-58 E6	5~31 (12)	
	35~84 (96)	37~85 (53)
	96~110 (10)	
	124~144 (17)	
HPV-58 E7	44~60 (3)	
	78~91 (7)	

Note: The column of “Distribution of HLA-I epitopes (epitope’s number)” refers to distribution of predicted HLA-I-restricted epitopes on the reference sequence and the number of predicted epitopes in each segment. The column of “Distribution of HLA-II epitopes (epitope’s number)” refers to distribution of predicted HLA-II-restricted epitopes on the reference sequence and the number of predicted epitopes in each segment

Prediction of HLA-II restricted epitopes used to further selection of optimal HLA-I restricted epitopes. We selected 46 HLA-II-restricted epitopes from the HPV-33 E6 reference sequence (see Additional file 3: Table S7) and then integrated the predicted epitopes without mutation sites into continuous segments. All of these epitopes appeared in the 38–73 segment (Table 6). For the HPV-33 E7 reference sequence, we selected 50 HLA-II-restricted epitopes (see Additional file 3: Table S8). The predicted epitopes without mutation sites were concentrated in the 3–25 and 46–96 segments (Table 6).

Finally, the top 5 (sorted by PR) optimal HLA-I-restricted epitopes in HPV-33 E6 and E7 were selected from the common segment with a concentrated distribution of HLA-I and HLA-II restricted epitopes. If no common segment was present, the optimal epitopes were selected based on prediction results of HLA-I. In this step, if a peptide was contained in another longer segment and the PR values were equal, we would choose the longer segment. If there were more predicted epitopes that have the same PR as the fifth selected, these epitopes would be included. Following this principle, we selected 5 and 6 optimal epitopes from HPV-33 E6 and E7, respectively (Table 7). It is valuable to determine whether a binding core of the MHC-II epitope exists in a MHC-I epitope because the core region of the MHC-II binding peptide is located in the groove of MHC-II, which plays a key role in the binding. Thus, the core regions of the HLA-II epitopes present in these optimal HLA-I-restricted epitopes are presented in Table 7, and at least one core region of HLA-II-binding peptide exists in each of the optimal epitopes selected from HPV-33 E6.

**Table 7:** Optimal predicted HLA-I-restricted epitopes selected from HPV-33 E6 and E7

Target Protein	Start	Length	Peptide	Percentile Rank	Binding Core in HLA-II predicted epitopes
HPV-33 E6	41	14	EVYDFAFADLTVVY	0.1	YDFAFADLT/DFAFADLTV/FAFADLTVV
	42	14	VYDFAFADLTVVYR	0.1	YDFAFADLT/DFAFADLTV/FAFADLTVV
	43	14	YDFAFADLTVVYRE	0.1	YDFAFADLT/DFAFADLTV/FAFADLTVV
	44	14	DFAFADLTVVYREG	0.1	DFAFADLTV/FAFADLTVV
	45	14	FAFADLTVVYREGN	0.1	FAFADLTVV
HPV-33 E7	52	10	YYIVTCCHTC	0.6	YYIVTCCHT
	59	8	HTCNTTVR	0.6	
	77	8	RTIQQLLM	0.1	
	77	14	RTIQQLLMGTVNIV	0.5	
	81	10	QLLMGTVNIV	0.55	
	82	9	LLMGTVNIV	0.4	

Note: The column of “Binding Core in HLA-II predicted epitopes” refers to the binding core of predicted HLA-II epitopes present in optimal HLA-I epitopes

#### T cell epitope prediction for HPV-58 E6 and E7

In total, 153 and 154 HLA-I-restricted epitopes were selected from the HPV-58 E6 reference and variant sequence, respectively (see Additional file 4: Table S9). By analysing the effect of mutations on epitopes, we found that substitution of D86E resulted in the PR of _81-92_YSLYGDTLEQTL for HLA-C*08:01 changing from 0.3 to 0.9, and substitution of D86E and K93 N led to the disappearance of _82-93_SLYGDTLEQTLK for HLA-A*11:01, which has a lower PR (0.3) in the reference sequence. All of the predicted epitopes without mutation sites were integrated into four different segments, which was similar to the integration results of HPV-33 E6 (Table 6). Most of the ideal epitopes with lower PR were included in the 35–84 segment. For HPV-58 E7, there are 18 and 24/22 potential epitopes selected from reference and variant-1/variant-2 sequences, respectively (see Additional file 4: Table S10). Analysis of results containing mutation sites revealed that a new potential epitope _7-20_TLKEYILDLHPEPI for HLA-A*02:01 appeared due to R9K and T20I, and the PR of _11-23_YILDLHPEPTDLF for HLA-C*01:02 changed from 0.5 to 0.9 due to T20I. Integration of predicted epitopes without mutation sites showed that these epitopes were concentrated in the 44–60 and 78–91 segments, which was also similar to the integration results of HPV-33 E7 (Table 6).

We also performed HLA-II-restricted epitope prediction for HPV-58 E6 and E7 reference sequences, and 67 and 35 predicted epitopes were selected from E6 and E7 reference sequences, respectively (see Additional file 4: Table S11 and Table S12). For HPV-58 E6, all predicted epitopes without mutation sites were found in the 37~85 segment (Table 6). For HPV-58 E7, due to the existence of 10 non-synonymous mutations, at least one non-synonymous mutation site was present in each predicted HLA-II-restricted epitopes. Following the principle of optimal epitope selection mentioned in Section 3.3.1, 7 and 6 optimal HLA-I-restricted epitopes were selected for HPV-58 E6 and E7, respectively, and six of the seven optimal epitopes from HPV-58 E6 had core regions of the HLA-II-binding peptide (Table 8).

**Table 8:** Optimal predicted HLA-I-restricted epitopes selected from HPV-58 E6 and E7

Target protein	Start	Length	Peptide	PercentileRank	Binding Core in HLA-II predicted epitopes
HPV-58 E6	40	11	SEVYDFVFADL	0.2	EVYDFVFAD
	41	11	EVYDFVFADLR	0.2	EVYDFVFAD
	44	12	DFVFADLRIVYR	0.2	DFVFADLRI/FVFADLRIV
	45	10	FVFADLRIVY	0.1	FVFADLRIV
	47	14	FADLRIVYRDGNPF	0.2	IVYRDGNPF
	61	12	AVCKVCLRLLSK	0.1	CKVCLRLLS
	71	13	SKISEYRHYNYSL	0.1	
HPV-58 E7	49	12	ATANYYIVTCCY	0.5	
	78	8	RTLQQLLM	0.2	
	78	14	RTLQQLLMGTCTIV	0.4	
	79	13	TLQQLLMGTCTIV	0.5	
	82	10	QLLMGTCTIV	0.45	
	83	9	LLMGTCTIV	0.4	

Note: The column of “Binding Core in HLA-II predicted epitopes” refers to the binding core of predicted HLA-II epitopes present in the optimal HLA-I epitopes

### B cell epitope prediction

#### B cell epitope prediction for HPV-33 E6 and E7

Epitope prediction results for B cells in HPV-33 are shown in Additional file 5: Table S13 and Table S14. In HPV-33 E6, a total of 16 B cell potential epitopes were discovered in the reference sequence, and 15 were identified in variant sequences. The three best epitopes of E6 were _51-66_TVVYREGNPFGICKLC, _124-139_RFHNISGRWAGRCAAC, and _3-18_QDTEEKPRTLHDLCQA. In HPV-33 E7, 10 B cell epitopes were predicted from both reference and variant sequences. The three best epitopes of E7 were _56-71_TCCHTCNTTVRLCVNS, _2-17_RGHKPTLKEYVLDLYP, and _48-63_ATADYYIVTCCHTCNT.

Amino acid mutations result in differences in HPV-33 E6 and E7 B cell epitopes predicted results between reference and variant sequences. In HPV-33 E6, K93N reduced the score of _89-104_EQTVKKPLNEILIRCI; _83-98_VYGNTLEQTVKKPLNE, _76-91_YRHYNYSVYGNTLEQT, and _98-113_EILIRCIICQRPLCPQ were reduced due to N86H and Q113R. In HPV-33 E7, S29T increased the score of _25-40_YEQLSDSSDEDEGLDR but decreased the score of _16-41_YPEPTDLYCYEQLSDS. The best potential epitope _31-46_SSDEDEGLDRPDGQAQ in E7 disappeared due to A45E/A45V.

#### B cell epitope prediction for HPV-58 E6 and E7

Epitope prediction results for B cell in HPV-58 are shown in Additional file 6: Table S15 and Table S16. A total of 13 B cell potential epitopes were discovered in both the E6 reference and variant sequences. The three best epitopes were _70-85_LSKISEYRHYNYSLYG, _34-49_KKTLQRSEVYDFVFAD, and _125-140_FHNISGRWTGRCAVCW. A total of 11 B cell potential epitopes were identified in both the E7 reference and variant sequences. The two best epitopes of E7 were _43-58_DGQAQPATANYYIVTC and _81-96_QQLLMGTCTIVCPSCA. Amino acid mutations account for the difference in HPV-58 E6/E7 B cell epitopes between reference and variant sequences. For example, in HPV-58 E6, the score of _81-96_YSLYGDTLEQTLKKCL decreased due to D86E, whereas in E7, the score of _33-48_DEDEIGLDRPDGQAQP increased from 0.86 to 0.91 due to G41R.

### Common segments of T cell and B cell epitopes

**Fig. 1:**
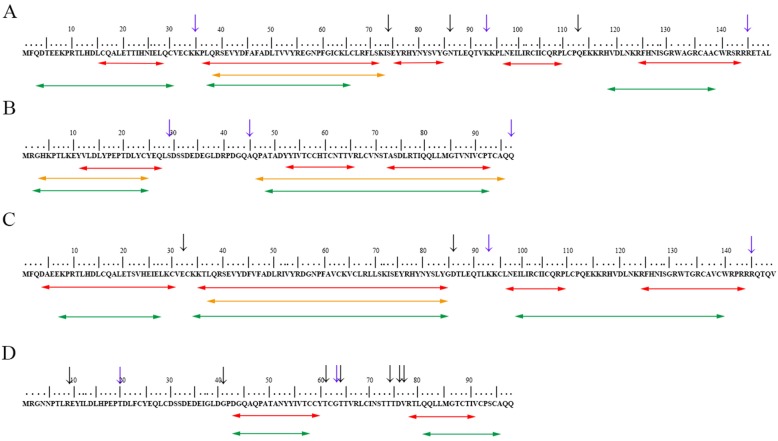
Distribution of predicted T cell and B cell epitopes. **a** reference sequence of  HPV-33 E6 protein. **b** reference sequence of  HPV-33 E7 protein. **c** reference sequence of HPV-58 E6 protein. **d** reference sequence of  HPV-58 E7 protein

Note: Red, yellow and green segments indicate a consensus of the predicted HLA-I, HLA-II and B cell epitopes, respectively, that were identical in reference and variant sequences. A black arrow indicates that this site is a non-synonymous mutation, and a purple arrow indicates that the corresponding site is a non-synonymous mutation and positive selection site simultaneously (positive selection sites were selected by Paml, indicating that mutation sites are evolutionarily competitive [19]). Numbers above the sequences are used to represent the position of residues.

In the study of T cell and B cell epitope prediction for HPV-33 and HPV-58 E6/E7, we found that amino acid substitution caused by nucleotide mutation influences the affinity of the antigen epitope, so we investigated the consistency of T cell epitopes and B cell epitope distribution in HPV-33 and HPV-58 E6/E7 (Fig. 1). There were mutual distributions of T cell and B cell epitopes on HPV-33 and 58 E6/E7 as well as the segments in which the above optimal T cell epitopes exists. These results further illustrated the importance of these segments in mediating immunogenicity.

## Discussion

HPV is an important pathogenic virus that is closely associated with diseases of the human skin and mucosa [25]. High-risk HPV infection causes 99.7% of cervical cancer [26]. HPV-33 and HPV-58 are highly homologous, and both are high-risk types in the α-9 genus. In addition, they were more prevalent and exhibit increased similarity in China compared with other areas and other types [2, 4, 22, 27]. HPV E6 and E7 oncoproteins are major virus transformation proteins that promote the development of cervical cancer; these oncoproteins are good targets for vaccine-induced cytotoxic T lymphocytes to prevent and treat carcinoma [21, 28]. Our research aimed at HPV-33 and 58 in Southwest China. Through polymorphism analysis, we identified some prevalent mutations that increase the risk of HPV carcinogenesis, such as T20I and G63S in HPV-58 E7. T20I is located near the Leu-Xaa-Cys-Xaa-Glu domain (residues 22–26) of E7. This domain mediates association with the retinoblastoma protein and its related proteins p107 and p130 [29]. G63S results in a change from glycine to serine. Previous studies have shown that the 31/32 serine residues of E7 are casein kinase II phosphorylation sites, which is important for the transformation of E7 [30]. Moreover, there is a positive association between phosphorylation rate and oncogenic potential [31]. Therefore, it is hypothesized that G63S may produce a new phosphorylation site and increase the carcinogenic risk of E7.

In order to select optimal epitopes for therapeutic vaccine design, we integrated bioinformatics techniques to investigate the influence of mutation on the secondary structure and epitope affinity. Polymorphism analysis demonstrated that multiple identical non-synonymous mutation sites exist in E6 of HPV-33 and 58, including the 86th, 93rd and 145th residue. The 93rd residue is the most common non-synonymous mutation in E6 of HPV-33 and 58, and both substitutions are from lysine to aspartate. Substitution of the 86th residue in HPV-33/58 E6 resulted in a change of secondary structure in the adjacent position, increasing the number of residues on the α helix after this site. In addition, based on previous studies in our lab, R145I in HPV-33 E6 and R145K in HPV-58 E6 are positive selection sites [17], and the core feature of positive selection sites is that the gene frequency of the variant increased rapidly and enhanced its adaptability to the environment. In our study, R145I in HPV-33 E6 completely abolished HLA-I- and HLA-II-restricted T cell epitopes containing this site, and the results are consistent with the conclusion that R145I is a positive selection site. It is hypothesized that substitution at this site can increase the gene frequency of the variant by reducing its immunogenicity and enhance its adaptability to the environment (R145K in HPV-58 E6 does not reflect this phenomenon mainly because the epitopes containing this site are not included in our selected epitopes based on the principle mentioned in the Materials and Methods section).

Accordingly, there are some significant differences between HPV-33 and HPV-58 in the epitope prediction results. DQB1*05:01 is considered to be a protective gene [32]. All mutations in HPV-33 E6/E7 did not affect the epitopes of DQB1*05:01. In contrast, the number of DQB1*05:01 epitope decreased from 10 to 5 in HPV-58 E6 due to D86E substitution, but increased from 10 to 12 in HPV-58 E7 due to T20I substitution. In addition to the above non-synonymous mutations, the predicted T cell epitopes are also affected by other non-synonymous mutations. For HPV-33 E6, the single mutations of K35N, N86H, K93 N and Q113R resulted in a general increase in epitope affinity. S74 T mutation or simultaneous mutation of N86H and K93 N caused some epitopes to decline while others increased. For HPV-33 E7, only A45V (A45E) appeared in the predicted epitopes, and the mutation causes a reduction in epitopes. Regarding HPV-58 E6, E32Q reduces the epitopes. In contrast, D86E and K93 N caused some epitopes to be reduced, while others increased. Regarding HPV-58 E7, the affinity of the epitopes containing both R9K and T20I mutations is significantly enhanced. D76E and V77A result in an increase in the predicted epitope number. Because these non-synonymous mutations have obvious influence on epitope affinity, we select optimal HLA-I-restricted epitopes for therapeutic vaccine design from prediction results without mutation sites combined with HLA-II-restricted epitope prediction results. All of the 6 optimal epitopes from HPV-33 E6 are located at _41-58_EVYDFAFADLTVVYREGN, and five of the seven optimal epitopes selected from HPV-58 E6 are located at _40-60_SEVYDFVFADLRIVYRDGNPF. These two segments have high sequence similarity and are ideal for designing therapeutic vaccines. Four of the six optimal epitopes selected from HPV-33 and 58 E7 correspond to the same position. Here, _77-84_RTIQQLLM, _77-90_RTIQQLLMGTVNIV, _81-90_QLLMGTVNIV and _82-90_LLMGTVNIV in HPV-33 E7 correspond to _78-85_RTLQQLLM, _78-91_RTLQQLLMGTCTIV, _82-91_QLLMGTCTIV, and _83-91_LLMGTCTIV in HPV-58 E7, respectively. These 4 optimal epitopes are located in _77-90_RTIQQLLMGTVNIV of HPV-33 E7 and _78-91_RTLQQLLMGTCTIV of HPV-58 E7. These two segments also exhibit high sequence similarity and are ideal for designing therapeutic vaccines. The above analysis reveals that most of the optimal epitopes from HPV-33 E6/E7 were located in the same segment as the optimal epitopes from HPV-58 E6/E7 and exhibit similar predicted affinity. This finding reflects the similarity of immunogenicity between HPV-33 and HPV-58.

## Conclusion

Nucleotide mutations in HPV-33 and HPV-58 *E6*/*E7* affect the composition and secondary structure of protein sequences and contribute to the affinity and immunogenicity of the peptide epitopes. In our study, optimal epitopes for therapeutic vaccines were selected from identical regions in reference and variant sequences. In addition, whether an epitope is included in the predicted results of HLA-II-restrictive epitopes is also considered. The final selections are based on the PR of predicted epitopes. The 6 optimal epitopes from HPV-33 E6 are located at _41-58_EVYDFAFADLTVVYREGN, and five of the seven optimal epitopes selected from HPV-58 E6 are located at _40-60_SEVYDFVFADLRIVYRDGNPF. The 4 optimal epitopes are located at _77-90_RTIQQLLMGTVNIV of HPV-33 E7 and _78-91_RTLQQLLMGTCTIV of HPV-58 E7. This information reflects the similarity of immunogenicity between HPV-33 and HPV-58. Using immune-informatics technologies to provide a new approach to access ideal epitopes, these results are valuable for the development of therapeutic vaccines and cancer immunotherapies.

## Additional files

Additional file 1: **Table S1.** Amino acid composition and hydrophilicity of HPV-33 E6 reference and variant sequence. **Table S2.** Amino acid composition and hydrophilicity of HPV-33 E7 reference and variant sequence. **Table S3.** Amino acid composition and hydrophilicity of HPV-58 E6 reference and variant sequence. **Table S4** Amino acid composition and hydrophilicity of HPV-58 E7 reference and variant sequence. (XLSX 16 kb)

Additional file 2: **Figure S1.** Secondary structure prediction of HPV-33 E6 reference and variant sequence. **Figure S2.** Secondary structure prediction of HPV-33 E7 reference and variant sequence. **Figure S3.** Secondary structure prediction of HPV-58 E6 reference and variant sequence. **Figure S4.** Secondary structure prediction of HPV-58 E7 reference and variant sequence. (PDF 754 kb)

Additional file 3: **Table S5.** The diversity of predicted binders of HPV-33 E6 for HLA-I alleles between reference and variant sequences; **Table S6.** The diversity of predicted binders of HPV-33 E7 for HLA-I alleles between reference and variant sequences; **Table S7.** Predicted epitopes binders for HLA-II alleles of HPV-33 E6; **Table S8.** Predicted epitopes binders for HLA-II alleles of HPV-33 E7. (XLSX 42 kb)

Additional file 4: **Table S9.** The diversity of predicted binder of HPV-58 E6 for HLA-I alleles between reference and variant sequences; **Table S10.** The diversity of predicted binders of HPV-58 E7 for HLA-I alleles between reference and variant sequences; **Table S11.** Predicted epitopes binders for HLA-II alleles of HPV-58 E6; **Table S12.** Predicted epitopes binders for HLA-II alleles of HPV-58 E7. (XLSX 42 kb)

Additional file 5: **Table S13.** Predicted linear B cell epitopes of HPV-33 E6; **Table S14** Predicted linear B cell epitopes of HPV-33 E7. (XLSX 13 kb)

Additional file 6: **Table S15.** Predicted linear B cell epitopes of HPV-58 E6; Table S16. Predicted linear B cell epitopes of HPV-58 E7. (XLSX 12 kb)

## Abbreviations

APC: Antigen-presenting cells
Bp: Base pair
CTL: Cytotoxic T lymphocyte
DNA: Deoxyribonucleic Acid
HLA: Human leukocyte antigen
HPV: Human Papillomavirus
HTL: Helper T lymphocyte
IEDB: Immune Epitope Database
MEGA: Molecular Evolutionary Genetics Analysis
MHC: Major histocompatibility complex
NCBI: National Center for Biotechnology Information
NK: Natural killer
PCR: Polymerase Chain Reaction
Rb: Retinoblastoma
SPSS: Statistical Product and Service Solutions
VLP: Virus-like Particles
