# Supplementary material for: Identification of variants and therapeutic epitopes in HPV-33/HPV-58 E6 and E7 in Southwest China
Source: Virol J. 2019 May 28;16:72. doi: 10.1186/s12985-019-1168-y (PMC6537359; doi:10.1186/s12985-019-1168-y)
Supplement: Supplementary file 2 — Figure S1. Secondary structure prediction of HPV-33 E6 reference and variant sequence. Figure S2. Secondary structure prediction of HPV-33 E7 reference and variant sequence. Figure S3. Secondary structure prediction of HPV-58 E6 reference and variant sequence. Figure S4. Secondary structure prediction of HPV-58 E7 reference and variant sequence. (PDF 754 kb) [file 12985_2019_1168_MOESM2_ESM.pdf]

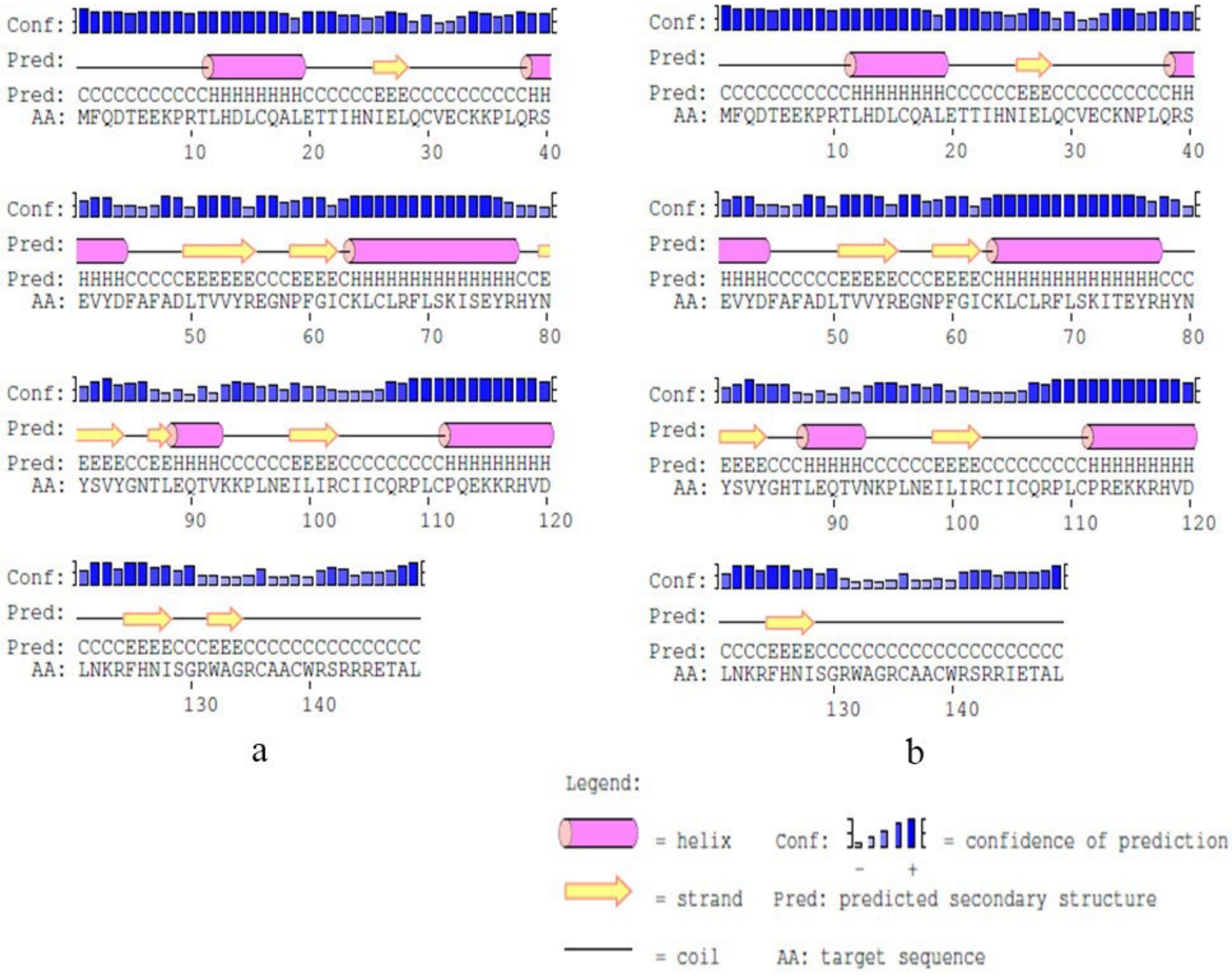

Figure S1. Secondary structure prediction of HPV-33 E6 reference and variant sequence

Note: a: secondary structure prediction model of HPV-33 E6 reference sequence; b: secondary structure prediction model of HPV-33 E6 variant sequence

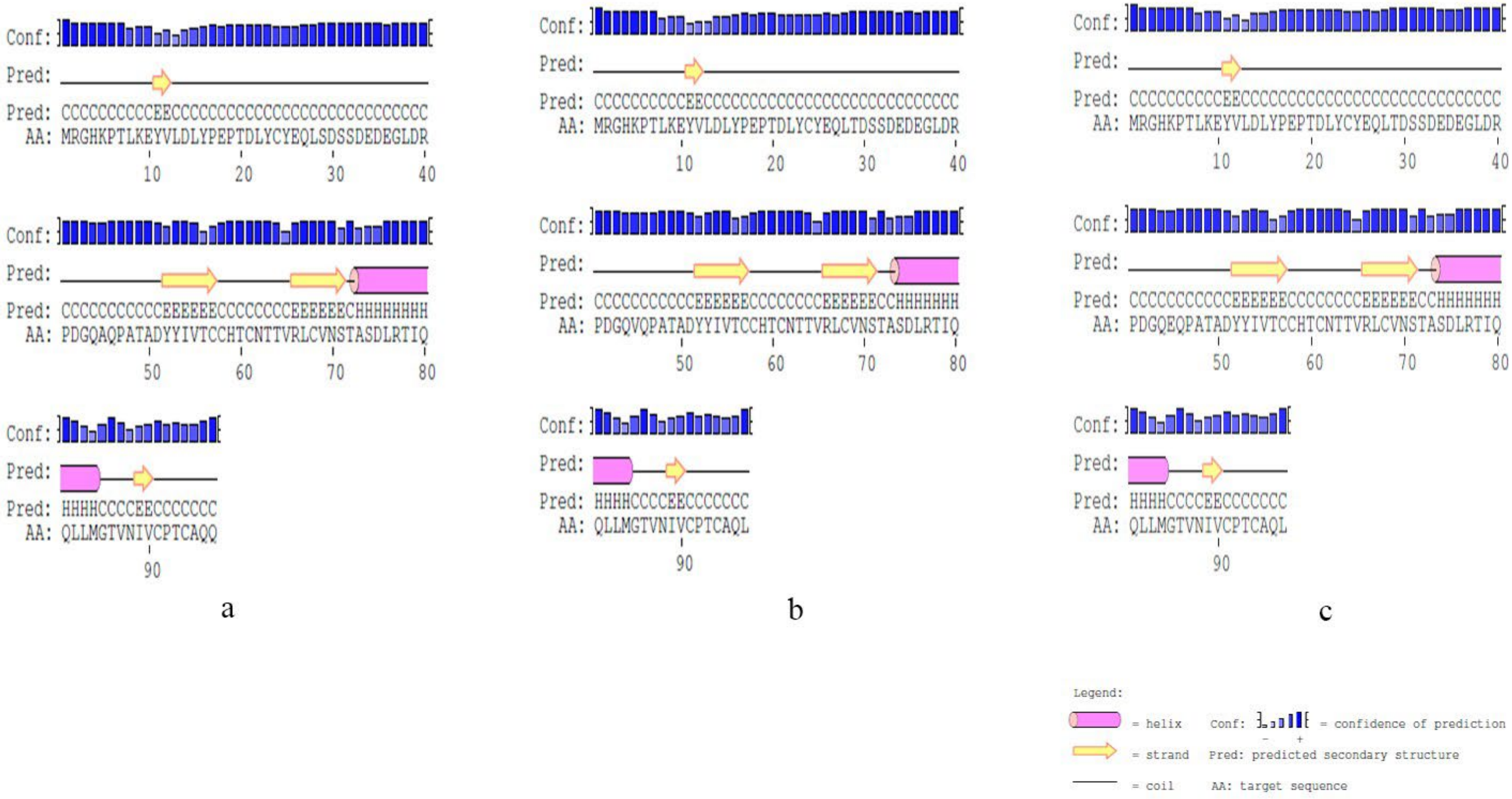

Figure S2. Secondary structure prediction of HPV-33 E7 reference and variant sequence

Note: a: secondary structure prediction model of HPV-33 E7 reference sequence; b: secondary structure prediction model of HPV-33 E7 variant-1 sequence; c: secondary structure prediction model of HPV-33 E7 variant-2 sequence

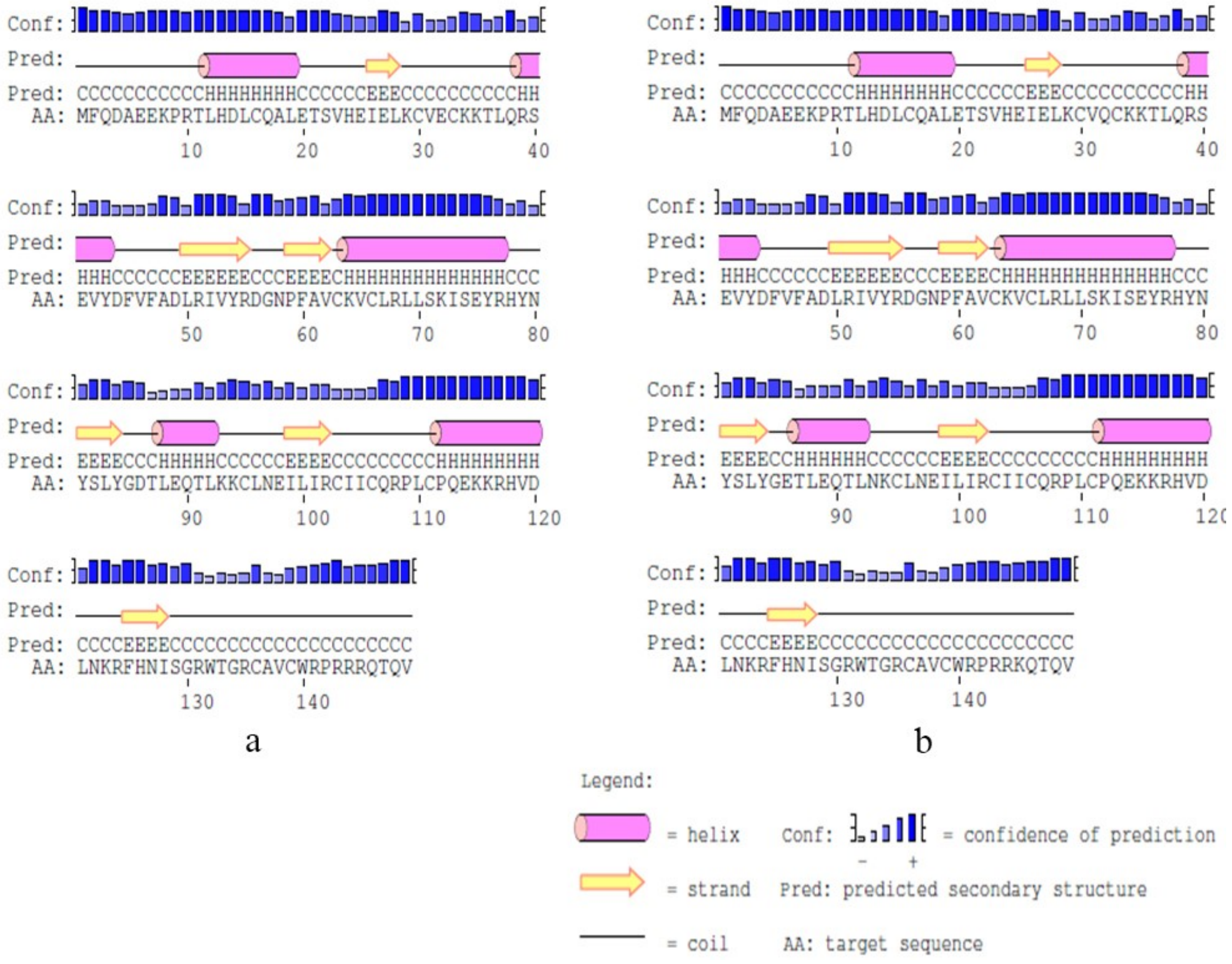

Figure S3. Secondary structure prediction of HPV-58 E6 reference and variant sequence

Note: a: secondary structure prediction model of HPV-58 E6 reference sequence; b: secondary structure prediction model of HPV-58 E6 variant sequence

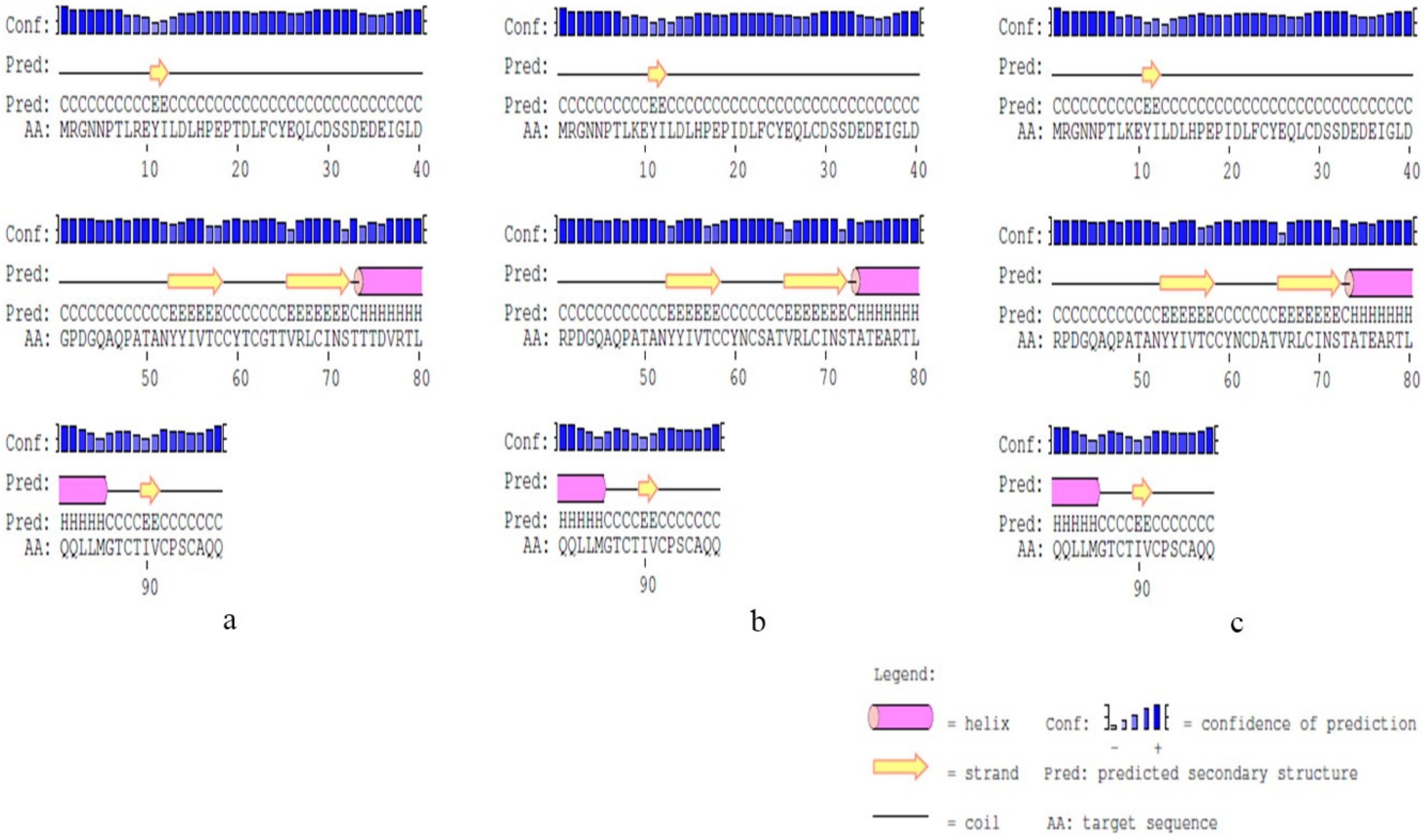

Figure S4. Secondary structure prediction of HPV-58 E7 reference and variant sequence

Note: a: secondary structure prediction model of HPV-58 E7 reference sequence; b: secondary structure prediction model of HPV-58 E7 variant-1 sequence; c: secondary structure prediction model of HPV-58 E7 variant-2 sequence
